# End-of-life care preferences of the general public and recommendations of healthcare providers: a nationwide survey in Japan

**DOI:** 10.1186/s12904-020-00546-9

**Published:** 2020-03-24

**Authors:** Jun Hamano, Kyoko Hanari, Nanako Tamiya

**Affiliations:** 1 Division of Clinical Medicine, Faculty of Medicine, University of Tsukuba, 1-1-1 Tennoudai, Tsukuba, 305-8575 Ibaraki Japan; 2 Graduate School of Comprehensive Human Sciences, University of Tsukuba, 1-1-1 Tennoudai, Tsukuba, 305-8575 Ibaraki Japan; 3 Faculty of Medicine, University of Tsukuba, 1-1-1 Tennoudai, Tsukuba, 305-8575 Ibaraki Japan

**Keywords:** End-of-life care, General public, Advanced cancer patient, Life-sustaining treatment, Nationwide survey, Hypothetical scenario

## Abstract

**Background:**

A better understanding of differences between the preferences of the general public and the recommendations of healthcare providers with regard to end-of-life (EOL) care may facilitate EOL discussion.

**Methods:**

The aim of this study was to clarify differences between preferences of the general public and recommendations of healthcare providers with regard to treatment, EOL care, and life-sustaining treatment (LST) based on a hypothetical scenario involving a patient with advanced cancer. This study comprised exploratory post-hoc analyses of “The Survey of Public Attitude Towards Medical Care at the End of life”, which was a population based, cross-sectional anonymous survey in Japan to investigate public attitudes toward medical care at the end of life. Persons living in Japan over 20 years old were randomly selected nationwide. Physicians, nurses, and care staff were recruited at randomly selected facilities throughout Japan. The general public data from the original study was combined to the data of healthcare providers in order to conduct exploratory post-hoc analyses. The preferences of the general public and recommendations of healthcare providers with regard to EOL care and LST was assessed based on the hypothetical scenario of an advanced cancer patient.

**Results:**

All returned questionnaires were analyzed: 973 from the general public, 1039 from physicians, 1854 from nurses, and 752 from care staff (response rates of 16.2, 23.1, 30.9, and 37.6%, respectively). The proportion of the general public who wanted “chemotherapy or radiation”, “ventilation”, and “cardiopulmonary resuscitation” was significantly higher than the frequency of these options being recommended by physicians, nurses, and care staff, but the general public preference for “cardiopulmonary resuscitation” was significantly lower than the frequency of its recommendation by care staff.

**Conclusion:**

Regarding a hypothetical scenario for advanced cancer, the general public preferred more aggressive treatment and more frequent LST than that recommended by healthcare providers.

## Key message

This nationwide cross-sectional survey investigated the preferences of the general public and recommendations of healthcare providers regarding end-of-life care based on the hypothetical scenario of an advanced cancer patient. The general public was found to prefer more aggressive treatment and more frequent life-sustaining treatment than that recommended by healthcare providers.

## Background

Personalized end-of-life (EOL) care is required for patients and their families to achieve a high-quality end of life [1], but 40–79% of terminally ill patients cannot express their own goals and preferences for medical treatment/care due to physical deterioration or mental incapacity [2–5]. Recent systematic reviews suggested that advanced care planning increases discussion about future treatment and EOL care, improves the quality of communication between patient and healthcare providers, and improves correspondence of the care provided with patient preferences in many patient populations [6, 7].

A recent international recommendation on advance care planning pointed out that involving multidisciplinary healthcare providers, including nurses and care staff, to support discussion about patient preferences regarding the goals of care is an important part of the EOL care process [8, 9].

Another recent study revealed a discrepancy between patient preferences for EOL care, including life-sustaining treatment (LST), and physician recommendations on EOL care [10, 11], but it is unclear whether differences also exist between patients and nurses or care staff with an essential role in providing EOL care.

As decisions about EOL care are often difficult and involve a high degree of discretion on the part of the healthcare provider, it is important for providers to understand that there may be differences between patient preferences and their recommendations about EOL care and LST.

A recent longitudinal study reported that EOL care was discussed between physicians and 9.2–18.3% of terminal cancer patients, and these discussions only increased significantly in the last month of life [12]. Barrio-Cantalejo et al. reported that 86% of older persons did not change their preferences for EOL care and LST [13]. Therefore, it may be important to investigate the preferences of the general public with regard to EOL care and LST in order to facilitate EOL care discussions between healthcare providers and terminal cancer patients.

Therefore, this study aimed to clarify differences between preferences of the general public and recommendations of healthcare providers (physicians, nurses, and care staff) with regard to cancer treatment, EOL care, and LST based on a hypothetical scenario involving a patient with advanced cancer. The secondary objective was 1) to explore factors influencing the preferences of the general public and the recommendations of physicians, nurses and care staff regarding cancer treatment, EOL care, and LST, and 2) to clarify the differences in recommendations regarding cancer treatment, EOL care, and LST among healthcare providers.

## Methods

### Participants and procedure

The aim of this study was to clarify differences between preferences of the general public and recommendations of healthcare providers with regard to treatment, EOL care, and life-sustaining treatment (LST) based on a hypothetical scenario involving a patient with advanced cancer.

This study comprised exploratory post-hoc analyses of “The Survey of Public Attitude Towards Medical Care at the End of life”, which was a population based, cross-sectional anonymous survey to investigate public attitudes toward medical care at the end of life conducted descriptively by the Ministry of Health, Labour and Welfare (MHLW), Japan in December 2017.

The source population of the original study was members of the general Japanese public aged 20 and over. The stratified two-stage random sampling method was used for selection. The survey was administered by mail in questionnaire packs. In addition to the questionnaire, the packs sent out by post also included explanatory letters from the MHLW, and stamped, addressed envelopes to return the questionnaire.

The participants of this study were members of the general public, physicians, nurses, and care staff. We used the general public data from the original study, and combined it with the data of healthcare providers in order to conduct exploratory post-hoc analyses, though the original study was conducted just for description not for analysis by the government. The questionnaire was sent by mail to 6000 members of the general public, 4500 physicians, 6000 nurses, and 2000 care staff, accompanied by a letter that concisely explained the survey. The details on how to select target participant and facilities, and how to distribute the questionnaire were described in our previous study [14]. The MHLW sent a reminder to all non-responders in January 2018. Completion and return of the questionnaire, in combination with the explanatory letter, was considered to indicate voluntary and informed consent to participate. The institutional review board of the University of Tsukuba approved the protocol of this study.

### Questionnaire

As there were no specific and validated instruments for evaluating the preferences of the general public and recommendations of healthcare providers regarding treatment, EOL care, and LST, we developed an original questionnaire based on data from previous studies [11, 15–17] and discussion among the authors of this study. Subsequently, we submitted the draft questionnaire to the MHLW and they made the final decision about the questionnaire items.

We used a scenario about a hypothetical cancer patient to investigate preferences for cancer treatment, EOL care, and LST based on the previous nationwide survey conducted in 2011 [15]. We submitted the draft questionnaire to the MHLW and they made the final decision about the questionnaire items. The respondents were asked to imagine that they were diagnosed with terminal cancer and going to die within 1 year (see Table 1), and were asked to express their preferences (or recommendations in the case of physicians, nurses, and care staff) about cancer treatment, EOL care, and LST. The treatments investigated were chemotherapy or radiation therapy for cancer (chemotherapy or radiation), fluid infusion if unable to drink water (fluid infusion), total parenteral nutrition (TPN) if unable to intake sufficient nutrition orally (TPN), nasogastric (NG) tube feeding if unable to intake sufficient nutrition orally (NG tube feeding), percutaneous endoscopic gastrostomy (PEG) tube feeding if unable to intake sufficient nutrition orally (PEG tube feeding), mechanical ventilation if it became difficult to breathe (ventilation), and cardiopulmonary resuscitation (CPR) if the heart or breathing stopped (CPR). The general public was asked to select from the following options: want treatment or care, do not want treatment or care, or not sure. For physicians, nurses, and care staff, the options were as follows: recommend treatment or care, do not recommend treatment or care, or not sure.

**Table:** **Table 1**

-Condition of the patient- After the diagnosis of terminal cancer, the condition has worsened. It has become difficult to eat and breathing is difficult. However, there is no pain, and consciousness and judgment are maintained at the same level as when healthy. -Medical judgment- “There is no prospect of recovery, and death will occur gradually or suddenly within approximately 1 year.”	

We collected the following background characteristics of the general public based on previous studies and discussion among the authors of this study: age in 5-year intervals from 20 to over 85, gender, living status, education, family doctor, and death of a close person within 5 years [11, 15]. We also collected the following background characteristics of the healthcare providers based on previous studies [11, 16, 17] and discussion among the authors: years of practice, workplace, frequency of caring for dying patients, identifying the proxy decision maker as standard practice, and participation in a nationwide education program on EOL discussion (binary category) [Palliative Care Emphasis Program on Symptom Management and Assessment for Continuous Medical Education (PEACE) and/or Education For Implementing End-of-Life Discussion (E-FIELD) for healthcare providers] [18–20]. PEACE is a 2-day interactive education program for physicians that integrates palliative care and psycho-oncology. It has been available since 2007. E-FIELD is a 1- or 2-day interactive education program for healthcare providers about respecting the patient’s wishes for EOL care and practicing based on the best interests of the patient, which has been held since 2016.

### Analysis

We initially conducted descriptive analyses of categorical variables. We defined “chemotherapy or radiation” as aggressive cancer treatment, “fluid infusion”, “NG”, “TPN”, and “PEG tube feeding” as EOL care, and “ventilation” and “CPR” as LST. We defined the answer “want/recommend treatment or care” as the preference for aggressive cancer treatment, EOL care and LST, and we defined “not sure” as missing data because it can be interpreted as both positive and negative opinions. We defined healthcare providers who answered “identify the proxy decision maker at some point (ex. when diagnosed as incurable disease or when death is approaching as the disease progresses)” as healthcare providers who identified the proxy decision maker.

Subsequently, we compared preferences about each type of cancer treatment, EOL care, and LST (seven in total) using the chi-square test or Fisher’s exact test. Lastly, we conducted multivariate logistic regression analysis of preferences for the seven treatments in each respondent group.

For logistic regression analysis of the general public, we used the following six independent variables based on previous reports and discussion among the authors of this study [15, 21, 22]: gender (reference: male), age <  64 years, education (reference: junior high or high school), living with family, death of a close person in the past 5 years, and presence of a family doctor.

For logistic regression analysis of healthcare providers, we used the following five independent variables based on discussion among the authors: working for > 30 years, working in hospital, caring for dying patients at least once a month, identifying the proxy decision maker, and participation in a nationwide education program. Probability values were based on two-sided tests and significance was accepted at *p* < 0.05. All analyses were conducted using SPSS-J (ver. 24.0; IBM, Tokyo, Japan).

## Results

A total of 973 members of the general public, 1039 physicians, 1854 nurses, and 752 care staff returned the questionnaire (response rates: 16.2, 23.1, 30.9, and 37.6% respectively). We analyzed all of the returned questionnaires. The characteristics of the respondents are summarized in Table 2. The respondents from the general public included 535 men (55.0%), and 448 were aged over 65 (46.1%). Among the healthcare providers, those working for > 31 years included 481 physicians (47.5%), 612 nurses (33.6%), and 29 care staff (3.8%). The most frequent workplace of doctors and nurses was hospitals [652 (64.4%) and 838 (45.9%), respectively], whereas it was long-term care facilities for the elderly in the case of care staff [396 (52.9%)].

**Table 2:** Characteristics of the respondents

General public						
		General public (*n* = 973)				
		n	%			
Gender	Male	535	55.0			
	Female	411	42.2			
Age	20–39	148	15.2			
	40–64	354	36.4			
	65–74	236	24.3			
	75-	212	21.8			
Education	Junior high school	109	11.2			
	High school	328	33.7			
	College	178	18.3			
	University/Graduate school	329	33.8			
Living with family	Yes	774	79.5			
Death of a close person in the past 5 years	Yes	391	40.2			
Family doctor	Yes	403	41.4			
Healthcare providers	Physicians (*n* = 1039)		Nurses (*n* = 1854)		Care staff (*n* = 752)	
	n	%	n	%	n	%
Years of practice						
1–15	139	13.4	317	17.1	386	51.3
16–30	392	37.7	895	48.3	334	44.4
31-	481	46.3	612	33.0	29	3.9
Workplace						
Hospital	652	62.8	838	45.2	n.a^b^	n.a^b^
Clinic	337	32.4	300	16.2	n.a^b^	n.a^b^
Long-term care facility	n.a^b^	n.a^b^	194	10.5	340	45.2
Care home	n.a^b^	n.a^b^	199	10.7	396	52.7
Visiting nurse office	n.a^b^	n.a^b^	210	11.3	n.a^b^	n.a^b^
Other	10	1.0	63	3.4	6	0.8
Participation in a nationwide training program	205	19.7	164	8.8	26	3.5
Frequency of caring for dying patients						
At least one patient per month	403	38.8	549	29.6	115	15.3
One patient per 6-months	230	22.1	631	34.0	349	46.4
One patient per year	131	12.6	270	14.6	200	26.6
Rarely	225	21.7	337	18.2	72	9.6
EOLD^a^ with patient						
To a sufficient extent	281	27.0	324	17.5	139	18.5
To some extent	385	37.1	809	43.6	280	37.2
Not much.	135	13.0	301	16.2	232	30.9
Not involved with dying patients	196	18.9	354	19.1	89	11.8
Identifying the proxy decision maker	830	79.9	1541	83.1	597	79.4
Sharing documented EOLD^a^ information with the multidisciplinary team	587	56.5	969	52.3	362	48.1

^a^*EOLD* End-of-life discussion

^b^*n.a* not applicable

### Comparison of the preferences of the general public and recommendations of healthcare providers regarding cancer treatment, EOL care, and LST

The highest preferences of the general public for cancer treatment, EOL care, and LST were as follows": "Fluid infusion" (48.5%), "Chemotherapy or radiation" (27.5%), and “TPN” (13.8%) (Additional file 1). Similarly, the most common recommendation about cancer treatment, EOL care, and LST made by physicians, nurses, and care staff was "Fluid infusion" (physicians: 59.5%, nurses: 56.4%, care staff: 53.6%) (Additional file 1).

**Fig. 1:**
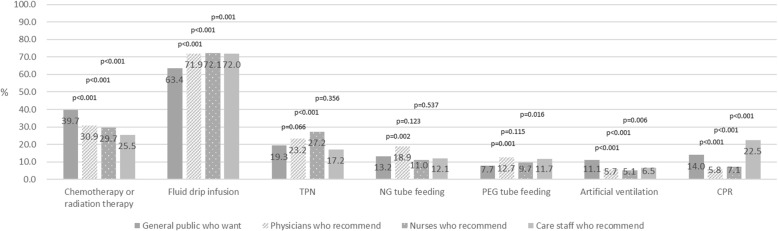
Comparison of end-of-life treatment and care preferences of the general public with recommendations of healthcare providers. This figure shows the preferences of the general public and recommendations of healthcare providers regarding cancer treatment, EOL care, and LST

The proportion of the general public who wanted “Chemotherapy or radiation” and “Ventilation” was significantly higher than the level of recommendation by physicians, nurses, and care staff (physicians: *p* < 0.001, nurses: *p* < 0.001, care staff: *p* = 0.006) (Fig. 1).

In addition, the proportion of the general public who wanted “CPR” was significantly higher than the level of recommendation by physicians and nurses (*p* < 0.001), although it was significantly lower than that by care staff (*p* < 0.001) (Fig. 1).

### Factors related to preferences of the general public and recommendations of healthcare providers with regard to cancer treatment, EOL care, and LST

Multivariate logistic regression analysis revealed that women among the general public had significantly lower preferences for “TPN”, “NG tube feeding”, and “CPR”. In addition, members of the general public over the age of 65 had significantly lower preferences for “TPN”, “NG tube feeding”, “PEG tube feeding”, “Ventilation”, and “CPR”. Other factors, such as education, living status, death of a close person in the past 5 years, and presence of a family doctor, did not have a significant relationship with preferences for cancer treatment, EOL care, and LST (Tables 3 and 4).

**Table 3:** Multivariate logistic regression analysis of end-of-life treatment and care preferences of the general public

	Gender (Ref = male)			Age (Ref = < 64 years old)		Education (Ref = Junior or high school)				Living with family			Death of close person in the past 5 years			Presence of family doctor		
	Exp (B)	95% CI	p	Exp (B)	95% CI	p	Exp (B)	95% CI	p	Exp (B)	95% CI	p	Exp (B)	95% CI	p	Exp (B)	95% CI	p
Chemotherapy or radiation therapy for cancer (*n* = 645)	0.73	0.52–1.01	0.055	0.70	0.48–1.03	0.067	1.15	0.80–1.64	0.455	0.71	0.45–1.11	0.129	1.18	0.85–1.64	0.314	1.01	0.70–1.45	0.950
Fluid infusion if unable to drink water (*n* = 714)	0.93	0.68–1.27	0.640	0.73	0.50–1.06	0.096	0.83	0.56–1.18	0.302	0.77	0.51–1.06	0.202	1.11	0.81–1.53	0.503	1.03	0.73–1.45	0.883
TPN^a^ if cannot intake sufficient nutrition orally (*n* = 657)	0.53	0.35–0.80	0.003	0.55	0.34–0.88	0.013	1.00	0.65–1.55	0.985	0.90	0.53–1.52	0.691	0.90	0.60–1.35	0.611	1.16	0.74–1.81	0.515
NG^b^ tube feeding if cannot intake sufficient nutrition orally (*n* = 680)	0.56	0.35–0.90	0.017	0.33	0.19–0.59	< 0.001	1.02	0.62–1.66	0.950	0.82	0.43–1.55	0.540	0.76	0.48–1.22	0.258	0.61	0.37–1.02	0.059
PEG^c^ tube feeding if cannot intake sufficient nutrition orally (*n* = 714)	0.66	0.38–1.16	0.152	0.40	0.20–0.80	0.009	0.72	0.40–1.31	0.279	0.79	0.36–1.74	0.566	1.53	0.88–2.67	0.132	0.90	0.49–1.67	0.740
Mechanical ventilation when it becomes difficult to breathe (*n* = 682)	0.65	0.40–1.08	0.094	0.29	0.15–0.55	< 0.001	0.87	0.51–1.49	0.616	1.19	0.64–2.21	0.579	1.01	0.61–1.66	0.981	0.89	0.51–1.55	0.672
Cardiopulmonary resuscitation if your heart or breathing stops (*n* = 745)	0.36	0.23–0.58	< 0.001	0.38	0.22–0.66	< 0.001	0.87	0.54–1.39	0.559	1.24	0.72–2.16	0.438	0.79	0.51–1.23	0.303	1.41	0.85–2.33	0.185

^a^*TPN* Total parenteral nutrition

^b^*NG* Nasogastric

^c^*PEG* Percutaneous endoscopic gastrostomy

**Table 4:** Multivariate logistic regression analysis of end-of-life treatment and care recommendations of healthcare providers

	Years of practice (Ref = under 30 years)			Working in a hospital			Caring for at least one dying patient per month			Identifying the proxy decision maker			Participated in a nationwide training program		
	Exp (B)	95% CI	p	Exp (B)	95% CI	p	Exp (B)	95% CI	p	Exp (B)	95% CI	p	Exp(B)	95% CI	p
Physicians															
Chemotherapy or radiation therapy for cancer (*n* = 734)	0.60	0.43–0.84	0.003	1.38	0.92–2.05	0.116	0.54	0.37–0.79	0.002	1.13	0.73–1.75	0.591	1.03	0.68–1.57	0.875
Fluid infusion if unable to drink water (*n* = 825)	0.77	0.56–1.06	0.105	1.49	1.01–2.22	0.047	0.45	0.31–0.66	< 0.001	2.00	1.34–2.96	0.001	0.65	0.43–0.97	0.033
TPN^a^ if cannot intake sufficient nutrition orally (*n* = 801)	0.99	0.70–1.39	0.941	1.66	1.10–2.50	0.016	0.48	0.32–0.72	< 0.001	1.22	0.76–1.97	0.412	0.51	0.31–0.84	0.008
NG^b^ tube feeding if cannot intake sufficient nutrition orally (*n* = 798)	1.29	0.89–1.86	0.182	2.01	1.28–3.14	0.002	0.52	0.34–0.80	0.003	0.71	0.44–1.13	0.145	0.51	0.30–0.87	0.014
PEG^c^ tube feeding if cannot intake sufficient nutrition orally (*n* = 819)	1.35	0.89–2.07	0.158	1.85	1.10–3.11	0.020	0.70	0.43–1.12	0.139	1.43	0.77–2.65	0.254	0.60	0.33–1.07	0.084
Mechanical ventilation when it becomes difficult to breathe (*n* = 848)	0.79	0.44–1.43	0.430	1.06	0.54–2.08	0.865	0.55	0.27–1.13	0.103	1.07	0.49–2.31	0.872	0.56	0.23–1.37	0.204
Cardiopulmonary resuscitation if your heart or breathing stops (*n* = 878)	1.08	0.59–1.96	0.812	2.38	1.14–4.96	0.021	0.50	0.25–0.98	0.044	1.32	0.56–3.09	0.527	0.23	0.07–0.75	0.015
Nurses															
Chemotherapy or radiation therapy for cancer (*n* = 1119)	1.08	0.82–1.43	0.586	1.57	1.19–2.07	0.002	0.41	0.30–0.57	< 0.001	0.88	0.61–1.27	0.501	0.74	0.44–1.26	0.268
Fluid infusion if unable to drink water (*n* = 1405)	0.69	0.53–0.89	0.005	2.03	1.56–2.64	< 0.001	0.38	0.29–0.50	< 0.001	0.70	0.48–1.00	0.052	0.39	0.26–0.58	< 0.001
TPN^a^ if cannot intake sufficient nutrition orally (*n* = 1307)	0.85	0.65–1.12	0.247	1.16	0.89–1.51	0.265	0.47	0.35–0.63	< 0.001	0.66	0.48–0.92	0.014	0.44	0.26–0.76	0.003
NG^b^ tube feeding if cannot intake sufficient nutrition orally (*n* = 1399)	0.71	0.49–1.04	0.078	1.29	0.90–1.84	0.163	0.34	0.21–0.54	< 0.001	0.51	0.34–0.77	0.001	0.64	0.30–1.35	0.238
PEG^c^ tube feeding if cannot intake sufficient nutrition orally (*n* = 1427)	0.86	0.59–1.27	0.446	1.86	1.28–2.70	0.001	0.26	0.15–0.43	< 0.001	0.47	0.31–0.72	< 0.001	1.03	0.52–2.05	0.937
Mechanical ventilation when it becomes difficult to breathe (*n* = 1436)	0.75	0.44–1.26	0.273	1.45	0.89–2.38	0.140	0.08	0.02–0.25	< 0.001	0.71	0.39–1.28	0.252	0.36	0.09–1.49	0.157
Cardiopulmonary resuscitation if your heart or breathing stops (*n* = 1462)	0.49	0.30–0.81	0.005	1.21	0.79–1.85	0.392	0.10	0.04–0.25	< 0.001	0.66	0.40–1.08	0.098	0.13	0.02–0.91	0.040
Care staff															
Chemotherapy or radiation therapy for cancer (*n* = 447)	0.28	0.06–1.22	0.090	1.60	1.03–2.49	0.036	1.00	0.55–1.80	0.997	1.16	0.66–2.04	0.615	0.97	0.34–2.78	0.959
Fluid infusion if unable to drink water (*n* = 560)	1.66	0.61–4.52	0.325	1.36	0.93–1.99	0.114	0.62	0.39–1.01	0.055	0.97	0.60–1.59	0.909	0.80	0.33–1.91	0.608
TPN^a^ if cannot intake sufficient nutrition orally (*n* = 499)	0.98	0.32–3.04	0.977	1.61	1.00–2.59	0.048	0.56	0.27–1.15	0.113	1.30	0.66–2.56	0.448	1.85	0.73–4.67	0.194
NG^b^ tube feeding if cannot intake sufficient nutrition orally (*n* = 539)	0.61	0.14–2.77	0.526	1.37	0.81–2.34	0.242	0.64	0.30–1.35	0.239	5.18	1.57–17.05	0.007	1.89	0.66–5.38	0.233
PEG^c^ tube feeding if cannot intake sufficient nutrition orally (*n* = 530)	0.43	0.09–2.16	0.306	2.30	1.31–4.06	0.004	0.90	0.42–1.90	0.774	2.31	0.94–5.64	0.067	6.18	2.51–15.23	< 0.001
Mechanical ventilation when it becomes difficult to breathe (*n* = 509)	2.11	0.58–7.72	0.260	1.81	0.87–3.78	0.111	0.00	0.00	0.997	0.88	0.38–2.06	0.770	1.21	0.26–5.64	0.810
Cardiopulmonary resuscitation if your heart or breathing stops (*n* = 515)	1.38	0.51–3.78	0.529	1.12	0.73–1.71	0.610	0.30	0.14–0.66	0.002	1.00	0.59–1.69	1.000	2.30	0.89–5.92	0.085

^a^*TPN* Total parenteral nutrition

^b^*NG* Nasogastric

^c^*PEG* Percutaneous endoscopic gastrostomy

Physicians who had participated in a nationwide training program were significantly less likely to recommend “Fluid infusion”, “TPN”, “NG tube feeding”, and “CPR”, whereas those working in hospitals were significantly more likely to recommend “Fluid infusion”, “TPN”, “NG tube feeding”, “PEG tube feeding”, and “CPR”.

Nurses who cared for at least one dying patient per month were significantly less likely to recommend all seven types of cancer treatment, EOL care, and LST, although nurses working in hospitals were significantly more likely to recommend “Chemotherapy or radiation”, “Fluid infusion”, and “PEG tube feeding”.

The care staff who worked in hospitals were significantly more likely to recommend “Chemotherapy or radiation”, “TPN”, and “PEG tube feeding”, whereas those who cared for at least one dying patient per month were significantly less likely to recommend “CPR” (Tables 3 and 4).

### Comparison of the recommendations regarding cancer treatment, EOL care, and LST among healthcare providers

Physicians were more likely to recommend NG tube feeding and PEG tube feeding than nurses, and were more likely to recommend chemotherapy or radiation therapy, TPN, and NG tube feeding than care staff.

Nurses were more likely to recommend TPN than physicians and care staff. Care staff had a higher rate of recommending CPR than physicians and nurses (Additional file 2).

## Discussion

This exploratory post-hoc analysis of a population based, cross-sectional anonymous survey in Japan revealed that the general public prefers more aggressive treatment and LST than that recommended by healthcare providers in a hypothetical advanced cancer patient scenario.

The first important finding was that the general public preferred more aggressive treatment and LST than that recommended by the healthcare providers, although CPR was less preferred compared with care staff recommendations. On the other hand, healthcare providers recommended limited medical care, i.e., life-prolonging care that promotes comfort and can be withdrawn, more often than was wanted by the general public [23]. These results suggest some dissonance between patients and healthcare providers with regard to aggressive treatment, LST, and limited medical care, which are considered to be quality indicators for EOL discussion [23–26]. The previous studies demonstrated, the general public has poor knowledge regarding the benefits of aggressive treatment and LST, such as CPR, parenteral nutrition, and hydration, for terminally ill cancer patients [27, 28]. Therefore, the general public might overestimate the success rate of CPR and the benefit of parenteral nutrition and hydration in late-stage cancer patients. A recent study found that specific information videos and websites regarding LST for the general public, such as the Talk CPR project, which provides knowledge regarding CPR [29], are helpful to reduce the dissonance between the general public and healthcare providers. Such information provision may also encourage patients and caregivers to approach their healthcare provider about this issue.

Our results are consistent with a previous study, which reported that physicians more often misinterpret the wishes of patients who do not want treatment [16]. Thus, healthcare providers should address the expectations of patients with advanced cancer about aggressive treatment, LST, and limited medical care to initiate and facilitate EOL discussion. A recent study suggested that providing specific information through different modalities can improve the confidence and understanding of healthcare providers regarding EOL discussion, and adding such information to existing education programs is considered effective [29].

The second important finding was that the workplace, participation in a nationwide education program on EOL discussion, and experience in EOL care were factors that significantly influenced the recommendations of healthcare providers about EOL care and LST.

Among physicians and nurses, working in a hospital was significantly positively associated with recommending fluid infusion, TPN, NG tube feeding, and PEG tube feeding. On the other hand, participating in a nationwide education program on EOL discussion and sufficient experience in EOL care were significantly negatively associated with recommending fluid infusion, TPN, NG tube feeding, and CPR.

Among the care staff, working at a long-term care facility had a significant positive association with recommending chemotherapy or radiation therapy for cancer, TPN, and PEG tube feeding, whereas having sufficient experience in EOL care had a significant negative association with recommending CPR.

It is difficult to change the workplace, but our study suggested that participation in a nationwide education program on EOL discussion and sufficient experience in EOL care can reduce dissonance regarding nutritional support among physicians, nurses, and patients.

The third important finding of this study was that the recommendations of each type of healthcare provider differed with regard to cancer treatment, EOL care, and LST. There were significant differences regarding the route of nutritional support recommended by physicians, nurses, and care staff, although they all recommended TPN most highly, followed by NG tube feeding and PEG tube feeding. Therefore, there was no major difference in the recommendations about nutritional support, but there were some differences in their preferences depending on the practice environment and individual experience.

Of note, care staff were significantly more likely to recommend CPR than physicians and nurses. This may reflect the anxiety of such staff about a patient dying in front of them, which was recently reported [30].

The fourth important finding was that members of the general public over 65 years old were significantly less likely to prefer NG tube feeding, PEG tube feeding, mechanical ventilation, and CPR. Moreover, female members of the general public were significantly less likely to prefer TPN, NG tube feeding, and CPR. This suggests that the preferences of patients can be inferred based on their demographics.

As this study was an exploratory post-hoc analysis, it has several limitations. First, we did not assess knowledge or beliefs about EOL care that may have influenced the preferences of the general public and the recommendations of healthcare providers about cancer treatment, EOL care, and LST. Second, our study was only conducted in Japan and the response rate was low; therefore, generalization of the results is difficult. Third, as respondents’ preparations for EOL care are different, our findings need to be interpreted with caution. However, regardless of these limitations, this study provided useful insights into the preferences of the general public and recommendations of healthcare providers with respect to cancer treatment, EOL care, and LST.

## Conclusion

The general public prefers more aggressive treatment and LST than that recommended by the healthcare providers in a hypothetical advanced cancer patient scenario, although the healthcare providers recommended EOL care more than preferred by the general public. Healthcare providers should ask the patient’s preference, taking into account that there may be differences between the patient’s preference and their recommendations regarding cancer treatment, EOL care, and LST.

## Supplementary information

**Additional file 1.** Preferences of the general public and recommendations of healthcare providers regarding EOL care and LST. Number and proportion regarding the preferences of the general public and recommendations of healthcare providers regarding EOL care and LST.

**Additional file 2.** Comparison of the recommendations regarding cancer treatment, EOL care, and LST among healthcare providers. Number and proportion regarding recommendations for cancer treatment, EOL care, and LST among healthcare providers.

## Data Availability

The datasets used and/or analyzed during the current study are available from the corresponding author on reasonable request.

## Abbreviations

CPR: Cardiopulmonary resuscitation
E-FIELD: Education For Implementing End-of-Life Discussion
EOL: End-of-life
LST: Life-sustaining treatment
MHLW: Ministry of Health, Labour and Welfare
NG: Nasogastric
PEACE: Palliative Care Emphasis Program on Symptom Management and Assessment for Continuous Medical Education
PEG: Percutaneous endoscopic gastrostomy
TPN: Total parenteral nutrition
