# Supplementary material for: End-of-life care preferences of the general public and recommendations of healthcare providers: a nationwide survey in Japan
Source: BMC Palliat Care. 2020 Mar 24;19:38. doi: 10.1186/s12904-020-00546-9 (PMC7093951; doi:10.1186/s12904-020-00546-9)
Supplement: Supplementary file 1 — Additional file 1. Preferences of the general public and recommendations of healthcare providers regarding EOL care and LST. Number and proportion regarding the preferences of the general public and recommendations of healthcare providers regarding EOL care and LST. [file 12904_2020_546_MOESM1_ESM.docx]

| Appendix 1 Preferences of the general public and recommendations of healthcare providers regarding EOL care and LST | | | | | | | | | | | | | | |  |  |  |  |  |  |  |  |  |  |
| --- | --- | --- | --- | --- | --- | --- | --- | --- | --- | --- | --- | --- | --- | --- | --- | --- | --- | --- | --- | --- | --- | --- | --- | --- |
|  | General public (n= 973) | | | | | | Physicians (n=1039) | | | | | | Nurses (n=1854) | | | | | | Care staff (n=752) | | | | | |
|  | want | | not want | | not sure | | recommend | | not recommend | | not sure | | recommend | | not recommend | | not sure | | recommend | | not recommend | | not sure | |
|  | n | % | n | % | n | % | n | % | n | % | n | % | n | % | n | % | n | % | n | % | n | % | n | % |
| Chemotherapy or radiation therapy for cancer | 268 | 27.5 | 407 | 41.8 | 225 | 23.1 | 234 | 22.5 | 523 | 50.3 | 225 | 21.7 | 346 | 18.7 | 819 | 44.2 | 603 | 32.5 | 114 | 15.2 | 333 | 44.3 | 289 | 38.4 |
| Fluid infusion if unable to drink water | 472 | 48.5 | 273 | 28.1 | 170 | 17.5 | 618 | 59.5 | 242 | 23.3 | 139 | 13.4 | 1045 | 56.4 | 407 | 22.0 | 337 | 18.2 | 403 | 53.6 | 157 | 20.9 | 182 | 24.2 |
| TPN* if cannot intake sufficient nutrition orally | 134 | 13.8 | 559 | 57.5 | 219 | 22.5 | 193 | 18.6 | 638 | 61.4 | 175 | 16.8 | 370 | 20.0 | 992 | 53.5 | 437 | 23.6 | 86 | 11.4 | 413 | 54.9 | 244 | 32.4 |
| NG^※^ tube feeding if cannot intake sufficient nutrition orally | 95 | 9.8 | 623 | 64.0 | 199 | 20.5 | 156 | 15.0 | 668 | 64.3 | 178 | 17.1 | 159 | 8.6 | 1291 | 69.6 | 347 | 18.7 | 65 | 8.6 | 474 | 63.0 | 204 | 27.1 |
| PEG^†^ tube feeding if cannot intake sufficient nutrition orally | 58 | 6.0 | 693 | 71.2 | 164 | 16.9 | 107 | 10.3 | 736 | 70.8 | 163 | 15.7 | 144 | 7.8 | 1333 | 71.9 | 326 | 17.6 | 62 | 8.2 | 468 | 62.2 | 215 | 28.6 |
| Mechanical ventilation when it becomes difficult to breathe | 79 | 8.1 | 634 | 65.2 | 200 | 20.6 | 50 | 4.8 | 830 | 79.9 | 125 | 12.0 | 76 | 4.1 | 1413 | 76.2 | 313 | 16.9 | 33 | 4.4 | 476 | 63.3 | 235 | 31.3 |
| Cardiopulmonary resuscitation if your heart or breathing stops | 110 | 11.3 | 673 | 69.2 | 131 | 13.5 | 53 | 5.1 | 857 | 82.5 | 97 | 9.3 | 108 | 5.8 | 1412 | 76.2 | 285 | 15.4 | 116 | 15.4 | 399 | 53.1 | 230 | 30.6 |

*TPN: Total parenteral nutrition

※NG: Nasogastric

†PEG : Percutaneous endoscopic gastrostomy
