# Supplementary material for: End-of-life care preferences of the general public and recommendations of healthcare providers: a nationwide survey in Japan
Source: BMC Palliat Care. 2020 Mar 24;19:38. doi: 10.1186/s12904-020-00546-9 (PMC7093951; doi:10.1186/s12904-020-00546-9)
Supplement: Supplementary file 2 — Additional file 2. Comparison of the recommendations regarding cancer treatment, EOL care, and LST among healthcare providers. Number and proportion regarding recommendations for cancer treatment, EOL care, and LST among healthcare providers. [file 12904_2020_546_MOESM2_ESM.docx]

Appendix 2 Comparison of the recommendations regarding cancer treatment, EOL care, and LST among healthcare providers

| Appendix 2-1 Physicians vs. nurses |  | Physicians | | Nurses | |  |
| --- | --- | --- | --- | --- | --- | --- |
|  |  | recommend | | recommend | |  |
|  |  | n | % | n | % | p-value |
| Chemotherapy or radiation therapy for cancer | (n=758, 1165) | 234 | 30.9 | 346 | 29.7 | 0.585 |
| Fluid infusion if unable to drink water | (n=860, 1461) | 618 | 71.9 | 1054 | 72.1 | 0.884 |
| TPN* if cannot intake sufficient nutrition orally | (n=831,1362) | 193 | 23.2 | 370 | 27.2 | 0.04 |
| NG^※^ tube feeding if cannot intake sufficient nutrition orally | (n=824, 1450) | 156 | 18.9 | 159 | 11.0 | <0.001 |
| PEG^†^ tube feeding if cannot intake sufficient nutrition orally | (n=843,1477) | 107 | 12.7 | 144 | 9.7 | 0.028 |
| Mechanical ventilation when it becomes difficult to breathe | (n=880,1489) | 50 | 5.7 | 76 | 5.1 | 0.545 |
| Cardiopulmonary resuscitation if your heart or breathing stops | (n=910,1520) | 53 | 5.8 | 108 | 7.1 | 0.219 |
|  |  |  |  |  |  |  |
| Appendix 2-2 Physician vs. care staff |  | Physicians | | Care staff | |  |
|  |  | recommend | | recommend | |  |
|  |  | n | % | n | % | p-value |
| Chemotherapy or radiation therapy for cancer | (n=758, 447) | 234 | 30.9 | 114 | 25.5 | 0.047 |
| Fluid infusion if unable to drink water | (n=860, 560) | 618 | 71.9 | 403 | 72.0 | 0.966 |
| TPN* if cannot intake sufficient nutrition orally | (n=831, 499) | 193 | 23.2 | 86 | 17.2 | 0.009 |
| NG^※^ tube feeding if cannot intake sufficient nutrition orally | (n=824, 539) | 156 | 18.9 | 65 | 12.1 | 0.001 |
| PEG^†^ tube feeding if cannot intake sufficient nutrition orally | (n=843, 530) | 107 | 12.7 | 62 | 11.7 | 0.585 |
| Mechanical ventilation when it becomes difficult to breathe | (n=880, 509) | 50 | 5.7 | 33 | 6.5 | 0.544 |
| Cardiopulmonary resuscitation if your heart or breathing stops | (n=910, 515) | 53 | 5.8 | 116 | 22.5 | <0.001 |
|  |  |  |  |  |  |  |
| Appendix 2-3 Nurses vs. care staff |  | Nurses | | Care staff | |  |
|  |  | recommend | | recommend | |  |
|  |  | n | % | n | % | p-value |
| Chemotherapy or radiation therapy for cancer | (n=1165, 447) | 346 | 29.7 | 114 | 25.5 | 0.095 |
| Fluid infusion if unable to drink water | (n=1461, 560) | 1054 | 72.1 | 403 | 72.0 | 0.936 |
| TPN* if cannot intake sufficient nutrition orally | (n=1362, 499) | 370 | 27.2 | 86 | 17.2 | <0.001 |
| NG^※^ tube feeding if cannot intake sufficient nutrition orally | (n=1450, 539) | 159 | 11.0 | 65 | 12.1 | 0.493 |
| PEG^†^ tube feeding if cannot intake sufficient nutrition orally | (n=1477, 530) | 144 | 9.7 | 62 | 11.7 | 0.205 |
| Mechanical ventilation when it becomes difficult to breathe | (n=1489, 509) | 76 | 5.1 | 33 | 6.5 | 0.237 |
| Cardiopulmonary resuscitation if your heart or breathing stops | (n=1520, 515) | 108 | 7.2 | 116 | 22.5 | <0.001 |
